# Analyzing of Gender Behaviors from Paths Using Process Mining: A Shopping Mall Application

**DOI:** 10.3390/s19030557

**Published:** 2019-01-29

**Authors:** Onur Dogan, Jose-Luis Bayo-Monton, Carlos Fernandez-Llatas, Basar Oztaysi

**Affiliations:** 1 Department of Industrial Engineering, Istanbul Technical University, Istanbul 34367, Turkey; odogan@itu.edu.tr (O.D.); oztaysib@itu.edu.tr (B.O.); 2 Instituto Universitario de Investigación de Aplicaciones de las Tecnologías de la Información y de las Comunicaciones Avanzadas (ITACA), Universitat Politècnica de València, Camino de Vera S/N, 46022 Valencia, Spain; jobamon@itaca.upv.es

**Keywords:** process mining, gender behavior, Bluetooth, indoor locations, shopping mall

## Abstract

The study presents some results of customer paths’ analysis in a shopping mall. Bluetooth-based technology is used to collect data. The event log containing spatiotemporal information is analyzed with process mining. Process mining is a technique that enables one to see the whole process contrary to data-centric methods. The use of process mining can provide a readily-understandable view of the customer paths. We installed iBeacon devices, a Bluetooth-based positioning system, in the shopping mall. During December 2017 and January and February 2018, close to 8000 customer data were captured. We aim to investigate customer behaviors regarding gender by using their paths. We can determine the gender of customers if they go to the men’s bathroom or women’s bathroom. Since the study has a comprehensive scope, we focused on male and female customers’ behaviors. This study shows that male and female customers have different behaviors. Their duration and paths, in general, are not similar. In addition, the study shows that the process mining technique is a viable way to analyze customer behavior using Bluetooth-based technology.

## 1. Introduction

Customer loyalty has critical importance for business continuity. The underlying problem is to understand customers. There are some research works on the relationship between customer loyalty and individualized proposals. For example, Infosys Company says companies that offer customer-oriented solutions have a big advantage concerning customer loyalty, such that 78% of consumers will be repeat customers [1]. As another example, Microsoft asserts that 73% of customers prefer companies that offer individualized purchasing solutions [2]. As a brand separator, customer experience will be more important than product and price by 2020 [3]. The research indicates the importance of understanding today’s customers having a digital purchasing experience.

Although individualized and customer-oriented solutions are more manageable for online companies, it is more difficult for the physical store because of the data collection about customers. On the other hand, according to the research by RetailNext [4], 84% of customers think that retailers should integrate the online and offline purchasing channels. Behavioral analytics can integrate online and offline channels for physical stores using different human tracking technologies [5,6,7,8]. RFID (radio frequency identification), WiFi (wireless fidelity), Bluetooth, and ZigBee are alternative technologies for customer tracking data. The technology is an essential factor affecting the customer relationship in physical stores. Since the customer behavior analysis (CBA) needs data to be gathered unobtrusively, the data should be captured using one of the indoor location system (ILS) sensors. In our previous study, we studied which data collection technologies were more appropriate for indoor customer tracking using the fuzzy multi-criteria decision-making technique [9]. As a result, we installed iBeacon devices, a Bluetooth-based data collection technology, in the shopping mall. Gathering data from customer’s smartphone connecting with Bluetooth provides the location and timestamp data belonging to the customer. Information can be generated about where the customers are in the shopping mall, the time they spend in each visited store, the next visit, etc. The created information also can be used for sales and marketing purposes [9,10].

Studying the understanding of indoor customer behavior concerning pathways is an interesting area [11]. In this study, we divide customer data as male and female customers to investigate whether there is a behavioral difference between male and female customers. We selected a shopping mall in Istanbul as an indoor area because retail sector employees are under high pressure due to inadequate professional training and human resources, a large number of operations, poor management, and poor customer-oriented service [12]. For that reason, better and more efficient use of resources is necessary for the improvement of the retail system and for strengthening customer relations. Performing simple statistics does not give general information about customers. One problem is a global view of the customers’ pathway to make the understanding of humans clear [13]. Besides, it is a requirement not only to discover a pathway followed by customers, but also to create an understandable output. Both discovery of pathways for a general view and generating understandable results are among the goals of process mining.

Process mining (PM) is a technique that aims to extract understandable information via process models generated automatically by different discovery algorithms from event logs using process management and business analytics [14,15]. A primary event log contains a case ID, activity, and timestamp data. The more data attributes such as resource, cost, etc., the more detailed the information about the process. A process model shows the flow of the data visually to make the process understandable. There can be different types of notations such as Petri nets, BPMN (business process modeling notation), and EPC (event-driven process chains). The process model is the output of the process discovery. Process mining has some discovery algorithms to generate process models automatically such as the alpha algorithm [16], fuzzy mining [17], heuristic mining [18], genetic mining [19], social network mining [20], inductive mining [10], and the PALIA suite (parallel activity log inference algorithm) [21].

Most of the pathway analyses use graphical representation to show the pathways. Without any detailed study, the created pathways are intuitive [10]. However, process mining explains the underlying information of the pathway. Therefore, process mining is selected to discover pathways of indoor customers to understand the behavior of customers visiting the shopping mall. In this study, behavioral analysis refers to understanding customer needs and proposes the best solutions to meet these needs. The study does not aim to investigate the behavioral science related to retail.

This paper is structured as follows. Firstly, studies related to customer behavior analysis and process mining are examined to show the importance of this study. Secondly, the selected algorithm and the proposed system are introduced in detail. Thirdly, the experimental case study is shown. Then, the Discussion and Conclusion Section is presented. Finally, the limitations of the study are discussed.

## 2. Related Works

There are many studies in the literature on behavior analysis to better understand people. It has many indoor implementations such as museums [22], hospitals [23,24], shopping venues [25,26], and airports [27]. Researchers have collected data in different ways. For example, Yoshimura et al. [22] took advantage of Bluetooth; on the other hand, Arroyo et al. [25] gathered data by recording with a camera.

Yoshimura et al. [22] used Bluetooth to determine the duration of the visitors stay at the Louvre Museum by taking into consideration the way of walking and the layout of the museum. As a result, museum visitors were categorized according to the duration of their stay. Oosterlinck et al. [5] showed the possibilities of using Bluetooth tracking in a shopping mall. They asserted that Bluetooth has a high data quality and low cost of data collection. Although camera technologies need complex image processing algorithms [5] and have poor performance because detecting people occurrences is not reliable [28], they are an excellent alternative to record every view of the customers. Merad et al. [6] performed purchasing behavior analysis according to the intensities of computer vision technology and customers in the store. Different algorithms were used to track the people in the video recordings. Wu et al. [7] created pathways of the customer in a retail store using a heat map.

Process mining has increasing and attractive importance in the literature. Efficiently collecting data through a wide variety of sensors provides for extracting valuable information about processes. Since healthcare has very dynamic and sophisticated processes [24], many researchers have concentrated on different healthcare processes. Rovani et al. [29] showed the differences between actual processes and guidelines described concerning the best-practices using conformance checking in the urology department. Mans et al. [30] applied process mining to dentistry. They first discovered the process model with the heuristic mining algorithm and then enhanced the discovery process. Fernandez-Llatas and colleagues [13] presented a process mining-based method using indoor location systems. With this method, data were gathered with RFID technology, without forcing nurses to measure time and other information about the processes, and entered into the systems. The data were used as input for the process mining, and the flows of the different processes were obtained. In another study, Fernández-Llatas et al. [31] used process mining to analyze the movements of people with 25 weeks of data belonging to nine people and collected by wireless technology.

Process mining has been applied to not only healthcare, but also municipalities [15,32], informatics [33,34,35], education [36,37,38], finance [39,40], and manufacturing [16,41].

We have realized from the literature review that data collected by different technologies are analyzed for various purposes. Table 1 shows some of them.

In our study, we used the collected data with Bluetooth to determine some descriptive statistics and discover pathways of the customers in the shopping mall.

Table 2 gives information about some studies. The PM column shows the studies collecting data with one of the technologies and creating pathways, but not focusing on customer behaviors. The study considers papers that collect data with one of the wireless or computer vision technologies such as RFID, WiFi, Bluetooth and camera in the literature review. Some papers used data obtained from IT systems such as Enterprise Resource Planning (ERP) [43] or social media such as Instagram [44]. Although these studies are not directly related to CBA, we include them in the literature review because they have valuable results.

There was only one paper focusing on customer behavior analysis using process mining and collecting data with a wireless sensor in an indoor location. Hwang and Jang [10] published a study that showed the possibilities of analyzing customer behavior using WiFi-based technology and the process mining technique. They used WiFi technology to gather customer data in a retail store in South Korea and applied inductive mining, one of the process mining discovery tools, to create customers’ trajectories. Although inductive mining removes infrequent activities and paths [54], it has some disadvantages. When the event log is incomplete or has exceptional data, the inductive miner is relatively sensitive [54]. In such cases, inductive miner algorithms cannot detect a strong relation in the event log. The second significant disadvantage of inductive mining is that it cannot deal with duplicate activities in the event log [55]. In some cases, to discover shoppers’ paths, activities with the same label can be observed in more than one location because beacons are generally located near each other in an indoor location to collect qualified data.

### Original Aspect of the Study

Although both PALIA suite and inductive miner algorithms produce process trees, PALIA suite has extra steps to tackle duplicate activities in the event log. It can combine nodes according to the name or importance. Importance between two nodes refers to the node names being the same, but there is a difference between them concerning spent time in the node. PALIA suite proposes two options: combine whatever it is or do not combine if it has a significant difference. On the other hand, the inductive mining algorithm aims to remove infrequent activities in the log to extract meaningful information. However, infrequent information can carry critical information about the daily movements of customers. For example, a visit to the cinema can be only once in a month, and according to the inductive mining view, these data should be removed from the dataset. Nevertheless, this visit may be critical for specifying a behavioral change of the customer. For example, not visiting the cinema can be a withdrawal symptom of timelessness or economic. PALIA suite can consider infrequent behavior with statistical information about the activities’ frequencies and state changes [31].

One of the main problems of the application of process mining techniques in environments with high variability is the appearance of the undesirable spaghetti effect problem [56]. The spaghetti effect is one of the most challenging problems in process mining studies [15]. It is related to the difficulty of the readability of sophisticated processes. Many transitions presented in the process model look like spaghetti. The high variability produced by the human behavior makes results complex to understand the flows inferred due to the appearance of all the possible movements that the users have. Furthermore, the objective of this work is not only to support professionals in a better understanding of the human behaviors at malls, but also to identify the different behaviors of different kinds of users. In recent works [57], clustering techniques have been applied to discover groups of behaviors. These techniques are based on the application of classic clustering algorithms such as k-means or quality threshold cluster, in combination with workflow distance measures [31], that group the most common behaviors. This combination allows, on the one hand, grouping common traces that have a similar behavior and, on the other hand, limiting the spaghetti effect because of the elimination of the variability in each one of the groups. PALIA suite has some clustering techniques implemented, allowing the discovery of different groups in an easy way. This tool has been used in real scenarios [57].

The other difference of our study from the study of Hwang and Jang [10] is the application area. Collecting data in only one retail store is more accessible than collecting in a shopping mall. At the same time, a shopping mall case requires more time than a store case for preprocessing steps and analyzing data. They collected data in a store; on the other hand, we collected data in one of the biggest shopping malls in Turkey, which has six floors and 293 stores.

On the other hand, selection of the data collection technology is also essential. When we compare WiFi and Bluetooth, Bluetooth technology outperforms WiFi technology concerning simplicity and accuracy criteria, while they have similar levels for time, cost, and bias [9]. At the same time, WiFi signals may have a problem with absorption [58]. Collecting comprehensive indoor data with WiFi is a big problem [59]. Bluetooth is a cost-effective and unannounced indoor tracking technology that provides unbiased and uninfluenced observations [46]. Furthermore, Bluetooth indoor tracking provides fast and accurate solutions [5]. According to Oosterlinck and coworkers [5], Bluetooth is a better alternative than WiFi because Bluetooth is more specifically designed for short ranges in indoor locations. Moreover, Bluetooth gives higher accuracy for an indoor location implementation. In summary, Bluetooth is a better data collection technology than WiFi in terms of indoor localization studies.

## 3. Methodology

To take advantage of the aggregated data, new technologies are needed to inform the whole process, making it easier for people to understand process characteristics. Process mining (PM) is a new way of using event logs to extract models that people can read [60]. Process mining uses event logs to discover, monitor, and improve processes. Event logs are the sum of the events considered as input for the process mining. In the Industrial 4.0 era, the digital world and the physical world are now intertwined. Today’s information systems hold event logs in extreme quantities. On the other hand, today’s technologies enable collecting much more data from smart devices. These opportunities make process mining a preferred technique to analyze collected data.

Process mining has three kinds of study topics: discovery, conformance checking, and enhancement [15]. The process discovery is the most important output of a process mining study [32]. Process discovery techniques use event logs to generate a process model without using any prior knowledge. The discovered model is represented by different notations such as Petri net and BPMN, which describe the behavior in the logs. There are several discovery algorithms such as heuristic mining [61], genetic mining [62], alpha [14], and PALIA suite [63]. In this study, we used PALIA suite [31].

**Figure 1:**
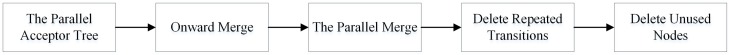
The parallel activity log inference algorithm (PALIA) suite steps.

PALIA suite is a discovery algorithm creating graph-based process models [13]. ILS data analysis is one of the implementation areas of graph-based process models that aims to present sophisticated information in a readable and understandable way [13]. PALIA is based on grammar inference theory, and in this case, it can be seen as a kind of Bayesian network with enhancement. Besides, PALIA keeps the traceability of all the real events that are related to each one of the nodes and transitions. Therefore, after discovery, researchers can recover the set of events related to each one of the nodes and transitions and even create new statistics. It consists of five main steps [21]. Figure 1 shows the steps of the PALIA suite.

- The parallel acceptor tree algorithm step produces process trees to start and end the time of events and their parallelism.
- The onward merge step fuses all branches of the tree. The algorithm checks for each node that the following two branches are equivalent. If so, they are fused. If all nodes and transitions use the same token for the same process in the two branches, it is said that the two branches are equivalent.
- The parallel merge step merges nodes that are sequential and represent the same event.
- The delete repeated transitions step deletes repeated transitions.
- The delete unused nodes step deletes unused nodes.

Finally, the algorithm produces a process model described as timed parallel automatons (TPA) that models the system. TPA represents a workflow as expressive as Petri nets [64].

For this study, we gathered data from customers visiting a shopping mall in Istanbul. The collected data contained infrequent paths typical of customer activity. For this reason, we needed a process mining algorithm that could cope with an infrequent and incomplete event log. PALIA suite can consider infrequent behavior with statistical information about the activities frequencies, and state changes [31]. In the case of the clustering algorithm, we selected a quality threshold cluster algorithm [65] that allows grouping by a similarity between the traces using a workflow distance [31] based on the error correcting paradigm [66] for limiting the spaghetti effect and maximizing the similarity among the traces.

## 4. iBeacon ILS

With the spread of the Internet of Things, beacon is a generic name given to small Bluetooth radio transmitters that offer personalized experiences. Beacon technology provides location information using Bluetooth Low Energy (BLE) or the trademarked name, Bluetooth^®^ Smart. Each iBeacon device has a coverage zone at a 50-m distance, and the interaction distance among devices can be fine-tuned in the zone. For that reason, one smart device can receive signals from more than one beacon device. In this case, proximity distances between the device and beacon are calculated and classified as near, medium, and far.

**Figure 2:**
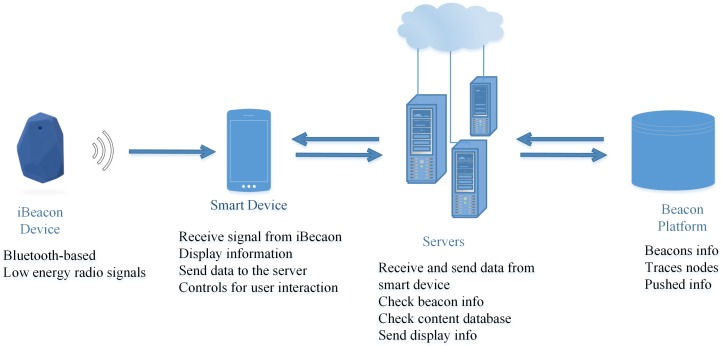
The architecture of beacon technology.

For the study, we collected data via iBeacon devices located in the shopping mall by a beacon network company, Blesh Company (Blesh.com) in Turkey. Approximately 300 iBeacon devices were located in the shopping mall for 293 stores by Blesh Company. Figure 2 depicts the main architecture of beacon technology and communication around this architecture. As a beacon network company, Blesh provides a software development kit (SDK) to third party application developers. Various third party mobile applications use this SDK to communicate with the beacon network [67]. In this way, the applications can provide an enhanced communication and value-added services to their customers through their own interface, after getting permission from the customers.

iBeacon devices transmit low energy radio signals and interact with the smart devices in their coverage zone. If the user allows, they automatically save the customer data, including a unique ID and time data. Each iBeacon device has a unique ID that represents a store. The system recognizes each smart device by its unique ID. Therefore, it allows us to create a pathway of the customer in the shopping mall. The beacon platform includes all beacons and location data. The server receives data from the smart device and contacts the beacon platform. Then, it sends display information to the smart device.

iBeacon ILS can produce CSV files that have in each line a positioning value for each event. Table 3 shows a sample part of the localization events provided by beacons. In each line, the following data are available:

- ID: For each captured datum, a unique number is defined to use different analysis. The ID column is not used in our study.
- Dongle columns: Because, in some cases, iBeacon devices are located near each other, one customer can be seen in three different locations. According to the proximity of the customer to the stores, iBeacon devices gather three different position data. Dongle_1 shows the nearest store where customer data were captured; on the other hand, Dongle_3 represents the farthest store location. In the study, we ignore Dongle_2 and Dongle_3 localization data.
- Timestamp: This is a date and time value that represents the moment at which data are captured by the iBeacon device for the related store. The timestamp format is dd.mm.yyyy hh:mm:ss.
- SubscriberID: This number shows the customer identification number associated with the mobile device.

Four basic perspectives are used in process mining [15] using the data attributes. The control-flow perspective considers the order of activities. The purpose of mining in this perspective is to find a good flow that represents all possible ways. The organizational perspective focuses on resources such as people, systems, roles, or departments in event records. Their relation to each other is examined. The aim is to show the social relations between resources or to classify resources regarding organizational unit and role. The event perspective (case perspective) focuses on the properties of events. An event can be represented by the process or by the initial activity of the event. For example, in a material replacement order, it may be important to know the supplier or to know the number of products ordered. The time perspective concerns the time or frequency of events. When the start and end times of the events are known, it is possible to determine the bottlenecks in the process, to measure the level of service, to view the use of resources, and to predict the remaining process.

## 5. Experimental Results

The data used in this research were collected in one of the major shopping malls in Istanbul. The shopping mall has six floors with a total of 293 shops. At the beginning of the study, 779,877 raw data were collected, which belonged to 12,000 unique customers. We grouped the stores concerning their products or services. For example, Store A, Store B, and Store C are grouped into the clothing shop group, if they sell mainly clothes. After some other data preparations, we used 642 customer data from which we could determine gender. Table 4 shows customer data details regarding the three months. In the collected data, there were 1293 cases that showed the number of customer paths in each visit. In these cases, 2749 store groups were visited. During these three months, at least one customer visited the shopping mall 52 times in a month. Because of the filter that we applied named as “shop split filter by gap”, the number of visits was higher than the total number of days in the month.

We hypothesize that the use of process mining algorithms provides the opportunity to understand indoor customer behaviors from their pathways in a human-understandable view and the behavioral difference based on gender. A detailed review of how customer behavior changes for behavioral science is not in the scope of this paper. One of the aims of this study is to show the potential of the combination of iBeacon ILS with process mining techniques.

To perform a primary process mining application, event identification, timestamp, and activity attributes are required from the event log. In our study to enhance the analysis, some additional data attributes were added to the corpus such as day number, day, day group, floor, and time interval.

### 5.1. Data Preparation

To apply process mining with the PALIA suite, firstly, we created an event log from collected ILS data. CustomerID, a unique identifier (ID), was created to track every customer. Location shows the stores in the shopping mall and was used to create customers’ pathways. The ILS dataset included a timestamp data. Instead of a timestamp, the PALIA suite requires a start time and an end time for each localization data. Therefore, we prepared our data by creating a start and end time from the timestamp. One customer can visit the shopping mall in different sessions. For example, customer X visits the shopping mall in the morning between 10:00 and 10:50. In the same day, the customer again visits the shopping mall from 19:30 to 21:00. These two visits are considered different sessions because the study aims to determine customer behavior. The customer may have different purposes during his/her visits. Moreover, since store group data are more meaningful and easy to interpret regarding the aim of the study, we also combined stores into store groups.

We applied some filters to extract more information. For example, the working time was restricted between 10:00:00 and 22:00:00 because of the opening time of the shopping mall. The shop duration filter adjusts the visit duration to be more than one minute. Otherwise, it is considered that the customer data were captured while walking instead of visiting. Therefore, a time gap occurred. Let the path (Store1—11:10:00), (Store2—11:14:00), (Store3—11:15:00), (Store1—11:22:00) show an event log belonging to the same customer. We can easily say that the customer visited Store1 at 11:10:00 and left at 11:13:59. Then, (s)he went to Store2. When we delete the Store2 event due to the one-minute filter, we encounter a time gap problem. The customer was in Store1 until 11:13:59 and then seen in Store3 at 11:15:00. Where is the customer between 11:13:59 and 11:15:00? This is a form of missing data. After the shop duration filter, we fused consecutive customer data called the fuse consecutive events filter to avoid this disappearance. The fuse consecutive events filter adjusts the ending time of Store1 visiting as 11:14:59, one second before the starting of the consequent event. The shop split filter by gap is applied if the time gap in between two subsequent observations in a path has a duration of 90 min; this is considered as a new visit. If an event is the last event in the trace, its end time is determined by adding one minute to its start time. Only the signals from the nearest location detected by iBeacon devices were assumed as the location where the customer was. The other signals were ignored and can be considered as missing data. In that case, we used Dongle_1 location data.

### 5.2. Process Mining

Although basic statistics do not give results on the general view of the customer pathways, they are useful to see some data-centric results.

Table 5 represents the size of data after preprocessing steps. For example, during the last month of 2017, we collected 290 customer data, 89 male data and 201 female data. In our study, we assumed that if one customer had a 90-min interval between two consecutive events, this was considered as a different visit. Therefore, during December, we had 450 different cases that referred to different visits by 290 customers. There was at least one customer that had 45 visit sessions.

**Figure 3:**
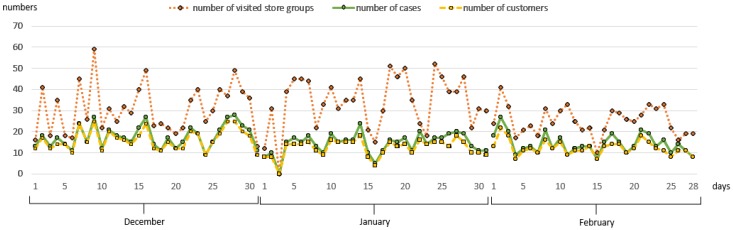
Number of customers and cases.

Figure 3 shows that there was a decrease in the last week of the month regarding the number of customers. Although the number of customers in December was more than the number of customers in January, customers in January visited more store groups than the other two months. At the same time, Figure 3 shows that some of the customers visited more than one time on the same day, resulting in inequality between the number of customers and number of cases.

Although the collected data did not contain gender information, it is clear that a customer going to the women’s bathroom was a woman and a customer going to the men’s bathroom was a man. In the figures showing customer paths, nodes and arrows are colored from green to red. The red nodes refer to the store group that had a higher visit duration. On the other hand, the red arrows present the most executed transaction between two nodes concerning the number of executions.

**Figure 4:**
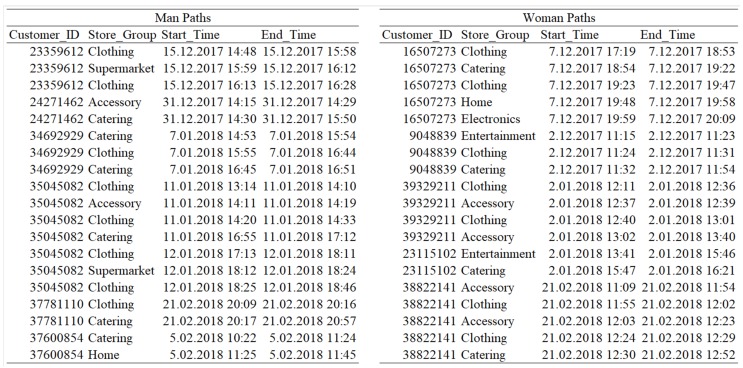
Sample validation data for discovered customer paths.

**Figure 5:**
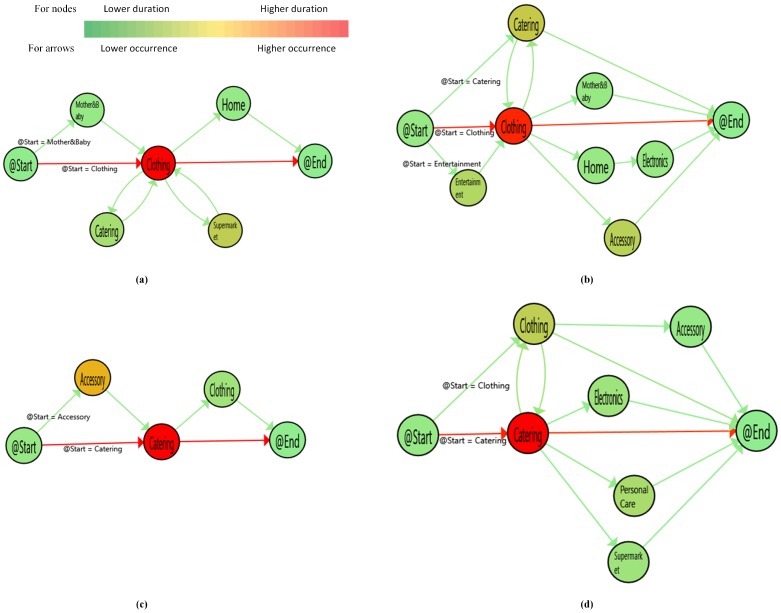
Customers’ paths in December. (**a**): Male paths-1st cluster (**b**): Female paths-1st cluster (**c**): Male paths-2nd cluster (**d**): Female paths-2nd cluster.

**Figure 6:**
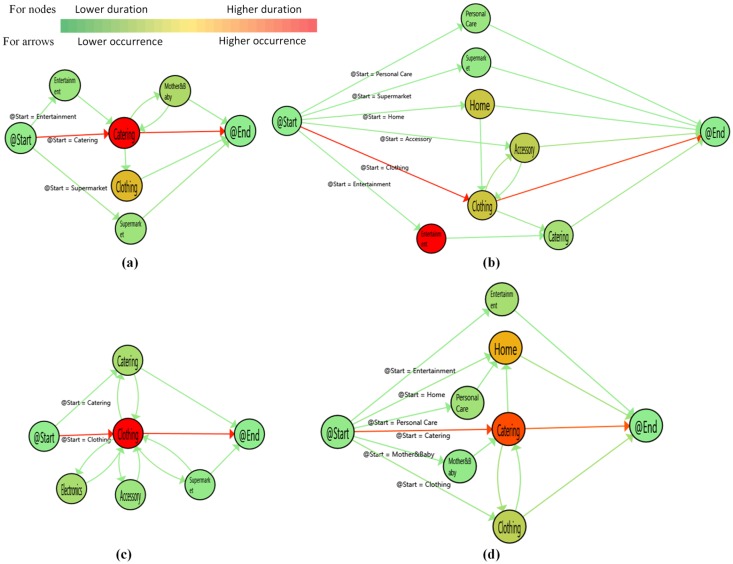
Customers’ paths in January. (**a**): Male paths-1st cluster (**b**): Female paths-1st cluster (**c**): Male paths-2nd cluster (**d**): Female paths-2nd cluster.

**Figure 7:**
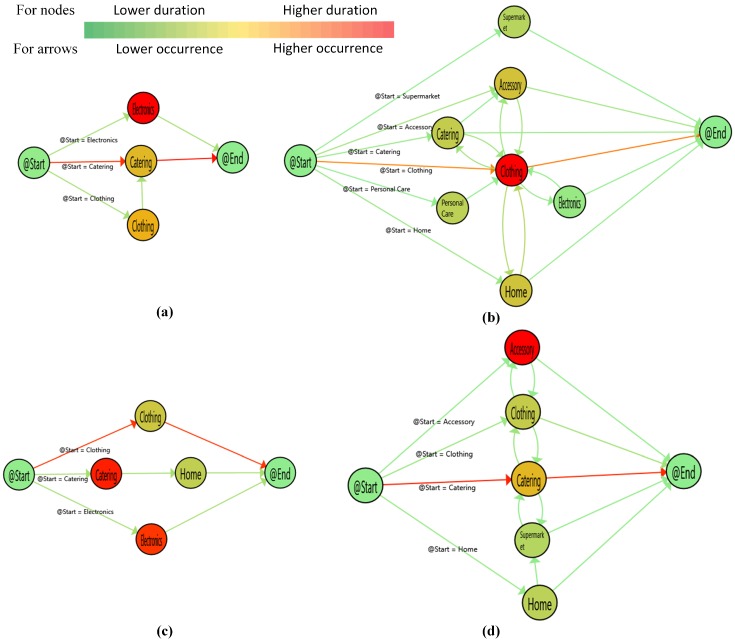
Customers’ paths in February. (**a**): Male paths-1st cluster (**b**): Female paths-1st cluster (**c**): Male paths-2nd cluster (**d**): Female paths-2nd cluster.

Since we clustered the customer paths with the PALIA suite to find meaningful and readable results, the path graphs do not represent all data in the event log. The graphs represent all male and female paths with rates of 0.824, 0.803 in December, 0.811, 0.835 in January, and 0.861, 0.824 in February, respectively for men and women. In the figure, (a) and (c) refer to mostly following male customer paths, and (b) and (d) refer to mostly following female customer paths. For example, Figure 5 includes two paths mostly followed by male and female customers in December. A sample of the data is given in Figure 4 to validate Figure 5, Figure 6 and Figure 7.

In December, catering and clothing were the most popular store groups in terms of the number of visits and duration (Figure 5). The numbers of customers who visited catering then clothing and vice versa were 16 and 14 in male customer visits and 35 and 47 in female customer visits, respectively. Male customers visited fewer store groups than female customers. Although accessory is a common store group, female customers spent less time there. Moreover, entertainment and personal care have significance concerning the behavioral difference. Male customers do not visit any entertainment and personal care stores in most followed paths.

When we compare December and January, female customers in January preferred entertainment instead of clothing (Figure 6). The entertainment duration was increased in January because some of the highly anticipated movies came out. Male customers still visited catering and clothing. Besides, electronics and mother and baby had a longer duration in male customer visits. Home showed another difference from December for female customers. The number visiting home was nearly similar, but the duration in home was four-times larger than the duration in December. It is interesting that, although female customers in Figure 6b mostly visited clothing, they spent more time in entertainment. In many cases, customers visiting entertainment visited a catering store to eat or drink.

Figure 7 represents February customer paths. Male customers mostly visited electronics and catering store groups. On the other hand, clothing and accessory store groups had the highest duration in female customer paths. Male customers usually spent their time in less visited places. For example, in Figure 7c, male customers mainly visited clothing and then left the shopping mall, but they mostly spent their time in catering and electronics.

## 6. Discussion and Conclusions

In this study, we present a process mining implementation to discover male and female customer paths in a shopping mall. This methodology has been tested using real three-month customer data from December 2017 to February 2018. The real data were collected using Bluetooth technology with iBeacon devices. Bluetooth is a cost-effective tracking technology that provides unbiased and uninfluenced observations. We used a process mining algorithm that could cope with an infrequent and incomplete event log. PALIA suite can consider infrequent behavior with statistical information about the activities’ frequencies and state changes. According to the results of the study, the implementation of process mining with ILS systems enables an understandable view. We evaluated results regarding basic statistics and process mining. While basic statistics give some data-centric results, process mining is process oriented. We investigated 642 customer paths, which was 165 male and 477 female customers. Since some customers visited the shopping mall more than one time, we created 1293 customer paths. The number of visited store groups was 2749 in this study. More customers visited the shopping mall in December, but more stores were visited in January. February was the weakest month with respect to the number of customers and visited stores. 9 December and 15 February were extremum dates. While 25 customers visited 59 store groups on 9 December, seven customers visited 10 store groups on 15 February.

In December, catering and clothing were the most popular store groups. They were the most visited and had the highest duration of visit among the store groups. The numbers of customers who visited catering then clothing and vice versa were 16 and 14 in male customer visits and 35 and 47 in female customer visits, respectively. Male customers had a loop between clothing-catering and clothing-supermarket. On the other hand, female customers had only a clothing-catering loop. That may mean that female customers are more decisive than male customers. We can also conclude that since male customers visited fewer store groups, they visited stores in an unplanned way. Male customers visited fewer store groups than female customers. Although accessory is a common store group, female customers spent less time there. Moreover, entertainment and personal care had significance concerning the behavioral difference. Male customers did not visit any entertainment or personal care stores in most followed paths.

In January, catering and clothing were the most popular store groups for male customers, as in December. On the other hand, the duration of visit in entertainment increased for female customers. The reason may be that some of the highly anticipated movies came out. Although female customers in Figure 7b mostly visited clothing, they spent more time in entertainment. In many cases, customers visiting entertainment visited a catering store to eat or drink. The loops in male customer paths were still higher than female customer paths, as in December. When we compared December and January, male customers had again visited catering and clothing. Besides, electronics and mother and baby had a greater duration in male customer visits. Home was another difference from December for female customers in January. The number visiting home was nearly similar, but the duration of visit in home was four-times larger than the duration in December.

In February, male customers preferred to visit electronic store groups instead of clothing. Catering was still one of the most visited locations. It is interesting that there was an inverse proportion between visited stores and duration in male paths. For example, male customers mostly visited catering in Figure 7a and clothing in Figure 7c, but spent more time in other store groups, electronics in Figure 7a and catering and electronics in Figure 7b. Contrary to the other two months, female customers had higher loops. Female customers visited accessory store groups instead of catering in this month. This may be because of Valentine’s Day. However, male customers did not become interested in giving a gift on that special day.

In a general manner, it is interesting that customers who spent more time in catering spent less time in clothing and vice versa. Figure 5, Figure 6 and Figure 7 support this idea. Although male and female customers had some similar traces, the duration was different. Catering had mainly the highest duration for male and female customers. However, some store groups such as entertainment and accessory became the critical points for behavioral changes in some cases.

We created a table that shows the number of visits and duration details in Table 6 to compare male and female customers’ behaviors. Number_Occurrence shows the total number of visits, Duration_Total refers to total duration in each store group, Duration_Average_By_Case presents the average duration with respect to the number of cases, and Duration_Average gives the average duration for each store group. Duration_Average_By_Case and Duration_Average are different because one store group may be visited more than one time in any case by the same customer. For example, male customers visited personal care eight times in December and spent one hour and 24 min. Since they visited personal care stores only one time in each case, Duration_Average_By_Case and Duration_Average were equal to 10 min and 29 s. On the other hand, female customers visited Personal Care 33 times in December and spent nine hours and 21 minutes. Since they visited personal care stores more than one time, at least one case, Duration_Average_By_Case, was 17 min and 31 s, and Duration_Average was 17 min.

**Figure 8:**
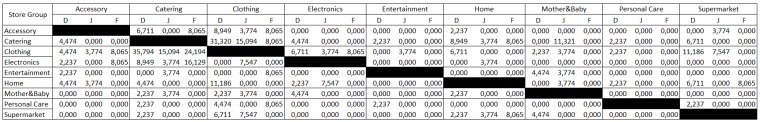
Probability matrix of transitions of male customers (×$10^{-3})$.

**Figure 9:**
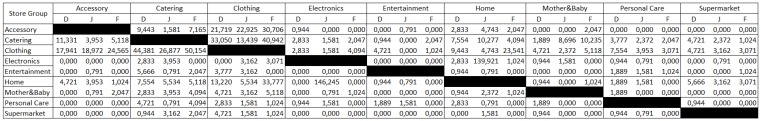
Probability matrix of transitions of female customers (×$10^{-3})$.

When we consider all paths for three months, Figure 8 and Figure 9 present the probability of all transitions among store groups. We realized exciting results from the matrix. All female customers visited a catering and clothing store each month. Home was visited by all female customers in December and January. Male customers visited entertainment after either catering or personal care in December, after clothing in January. Although catering and clothing are two similar store groups for male and female customers, the difference is that the possibility of visiting clothing after catering and catering after clothing was much higher in female visits than male visits. Male customers visited catering after clothing by a rate of 0.031 and clothing after catering by a rate of 0.036 during December, which had the highest transition probabilities. In the same month, female customers had rates of 0.033 and 0.044 for transition probability from catering to clothing and clothing to catering, respectively. In January paths, male customers visited personal care as only the first visit point. They had no transition from other store groups to personal care. On the other hand, female customers always visited personal care from other store groups except for accessory and mother and baby. Entertainment was a critical store group in January, especially for female customers. Female customers visited entertainment after accessory, home, and Personal Care by a probability of 0.001, 0.001 and 0.002. After visiting Entertainment, they mainly visit either Clothing by a rate of 0.003 or return personal care by a rate of 0.002. However, male customers visited entertainment after only one store group, clothing, by a rate of 0.004. Then, they continued to visit either catering or mother and baby with equal transition probability, 0.004.

## 7. Future Research and Limitations

We considered all customer data in primary screening. Then, we focused on male and female customer data to assess their behavior in secondary screening. As future work, personalized guidance may be developed for customers. Customer-specific suggestions or future state prediction may be considered. Furthermore, a process mining framework may be designed as in the study of Yang and Hwang [68]. They used process mining and introduced a framework to uncover fraud and abuse in healthcare. One of the advantages of process mining is to detect bottlenecks. Crowds in a shopping mall can be thought of as a bottleneck. Then, crowded store groups could be investigated. We have so much data in our corpus, that we do not know the gender. We defined genders according whether customers go to the men’s bathroom or women’s bathroom. For further research, we will predict the gender of customers from the paths. As training data, we will use the known data that we used in this study.

Because of the technical properties of beacon software, only three localization data were collected. We cannot manipulate them. We assumed the location where the customer visited was shown by the nearest dongle. This is one of our assumptions. The study may be expanded by considering medium and far location data.

Use of Bluetooth for tracking purposes is an applicable approach, but there are some limitations. For example, customers must allow data sharing with the Bluetooth devices because the operating system often limits the access to customer’s mobile devices. At the same time, although Bluetooth is a useful tracking technology, it is necessary to pre-install.

## Figures and Tables

**Table 1:** Purpose of the collected data.

Purposes	Studies
To determine some parameters (average number of places visited by people, the average time spent by them, most visited/least visited places) with descriptive statistics	[22]
To discover routes followed by customers	[7,22]
To estimate the next places visited	[42]
To determine where the customer is located at any time	[5,28]

**Table 2:** Summary of the literature review. CBA, customer behavior analysis; PM, process mining.

Study	CBA	Technology	PM	Implementation Area
[45]	✔	Bluetooth		Museum
[22]	✔	Bluetooth		Museum
[46]	✔	Bluetooth		Exhibition
[5]	✔	Bluetooth		Store/Shopping Mall
[47]	✔	Bluetooth		Hospital
[48]	✔	Bluetooth		Museum
[49]	✔	Bluetooth		Museum
[7]	✔	Camera		Store/Shopping Mall
[25]	✔	Camera		Store/Shopping Mall
[26]	✔	Camera		Store/Shopping Mall
[50]	✔	Camera		Store/Shopping Mall
[6]	✔	Camera		Store/Shopping Mall
[51]		RFID		Hospital
[52]	✔	RFID		Store/Shopping Mall
[53]	✔	RFID		Store/Shopping Mall
[8]	✔	RFID		Store/Shopping Mall
[10]	✔	WiFi	✔	Store/Shopping Mall
[31]	✔	RFID	✔	Hospital
[44]	✔	Other	✔	Exhibition
[43]	✔	Other		Manufacturing

**Table 3:** An example of an event log created from iBeacon ILS.

ID	Dongle_1	Dongle_2	Dongle_3	Timestamp	SubscriberID
1028333326	121527			11.12.2017 00:14:42	17399446
1028334382	121498			11.12.2017 00:16:48	39081930
1028334406	121498	121404		11.12.2017 00:16:50	39081930
1028334421	121498			11.12.2017 00:16:53	39081930
1028492822	121436	121510	121446	11.12.2017 07:23:47	29078632
1028492925	121510	121372		11.12.2017 07:23:59	29078632
1028492939	121436	121510	121446	11.12.2017 07:24:01	29078632
1028495185	121446	121436	121510	11.12.2017 07:28:23	29078632

**Table 4:** Summary of the corpus after combining into shop groups.

	Customer Data
Total Customer (Man/Woman)	642 (165/477)
Total Number of Cases	1293
Maximum Number of Visit Sessions	52
Localization Events	2749

**Table 5:** Basic statistics of the preprocessed data.

	December 2017	January 2018	February 2018
Total Customer (Man/Woman)	290 (89/201)	181 (47/134)	171 (29/142)
Total Number of Cases	450	444	399
Maximum Number of Visit Sessions	45	52	43
Localization Events	957	1088	704

**Table 6:** All data comparison.

	**Number_Occurrence**						**Duration_Total**					
	**December**		**January**		**February**		**December**		**January**		**February**	
Nodes	Man	Woman	Man	Woman	Man	Woman	Man	Woman	Man	Woman	Man	Woman
Accessory	14	62	4	66	4	81	02:41:59	1.03:05:59	00:25:59	1.20:10:59	02:42:59	3.01:53:59
Catering	79	200	53	123	26	154	2.03:18:59	4.12:33:59	1.15:48:59	3.07:43:59	10:27:59	3.16:57:59
Clothing	93	223	60	189	17	242	9.18:33:59	4.00:46:59	8.19:34:59	5.14:16:59	05:10:59	17.18:42:59
Electronics	23	16	6	217	10	12	12:39:59	05:22:59	02:22:59	4.17:05:59	07:54:59	02:26:59
Entertainment	6	20	4	19	1	10	02:08:59	14:21:59	01:47:59	09:15:59	00:20:59	01:40:59
Home	24	77	10	252	6	76	06:51:59	1.04:35:59	02:44:59	5.11:04:59	06:54:59	2.01:46:59
Mother and Baby	10	24	6	27	0	31	02:30:59	09:08:59	02:09:59	11:00:59	00:00:00	2.02:08:59
Personal Care	8	33	0	22	1	13	01:23:59	09:20:59	00:00:00	05:37:59	01:03:59	06:32:59
Supermarket	19	26	7	23	5	15	05:44:59	11:26:59	02:28:59	07:25:59	00:54:59	05:15:59
	**Duration_Average_by_Case**						**Duration_Average**					
	**December**		**January**		**February**		**December**		**January**		**February**	
Nodes	Man	Woman	Man	Woman	Man	Woman	Man	Woman	Man	Woman	Man	Woman
Accessory	00:13:29	00:29:33	00:06:29	00:47:20	00:40:44	01:04:15	00:11:34	00:26:13	00:06:29	00:40:09	00:40:44	00:54:44
Catering	00:44:37	00:35:24	00:46:50	00:42:20	00:24:09	00:37:19	00:38:58	00:32:34	00:45:04	00:38:53	00:24:09	00:34:39
Clothing	02:53:45	00:30:43	03:59:31	00:48:32	00:19:26	02:19:08	02:31:19	00:26:02	03:31:34	00:42:37	00:18:17	01:45:47
Electronics	00:34:32	00:20:11	00:23:49	02:41:34	00:47:29	00:12:14	00:33:02	00:20:11	00:23:49	00:31:16	00:47:29	00:12:14
Entertainment	00:25:47	00:45:22	00:26:59	00:30:53	00:20:59	00:10:05	00:21:29	00:43:05	00:26:59	00:29:15	00:20:59	00:10:05
Home	00:19:37	00:25:14	00:18:19	01:39:33	01:09:09	00:45:15	00:17:09	00:22:17	00:16:29	00:31:12	01:09:09	00:39:18
Mother and Baby	00:15:05	00:22:52	00:25:59	00:25:25	00:00:00	02:10:49	00:15:05	00:22:52	00:21:39	00:24:28	00:00:00	01:37:03
Personal Care	00:10:29	00:17:31	00:00:00	00:15:21	01:03:59	00:30:13	00:10:29	00:16:59	00:00:00	00:15:21	01:03:59	00:30:13
Supermarket	00:18:09	00:26:25	00:21:17	00:19:23	00:13:44	00:21:03	00:18:09	00:26:25	00:21:17	00:19:23	00:10:59	00:21:03
